# 

**Published:** 1853-12

**Authors:** 


At a Special Meeting of the Northern Medical Association of Philadelphia, held Nov. 15, 1853, the following preamble and resolutions were unanimously adopted:

*Whereas,* It has pleased Almighty God to remove from our midst to his final home, Dr. Thomas Hobson, one of our esteemed and talented associates, therefore

*Resolved,* That we deeply sympathise with his bereaved family in this dispensation of Divine Providence, and that the President and Secretary be instructed to convey to them the sincere condolence of this Association, for the irreparable loss they have sustained.

*Resolved,* That the members of this Association attend his funeral.

*Resolved,* That the foregoing preamble and resolutions be published in the Medical Examiner and Medical News, and in one of the daily papers.

N. L. Hatfield, President.

Thos. Bond, Secretary.

